# Supplementary figures and images for: The reanalysis of biogeography of the Asian tree frog, Rhacophorus (Anura: Rhacophoridae): geographic shifts and climatic change influenced the dispersal process and diversification
Source: PeerJ. 2017 Nov 21;5:e3995. doi: 10.7717/peerj.3995 (PMC5701547; doi:10.7717/peerj.3995)

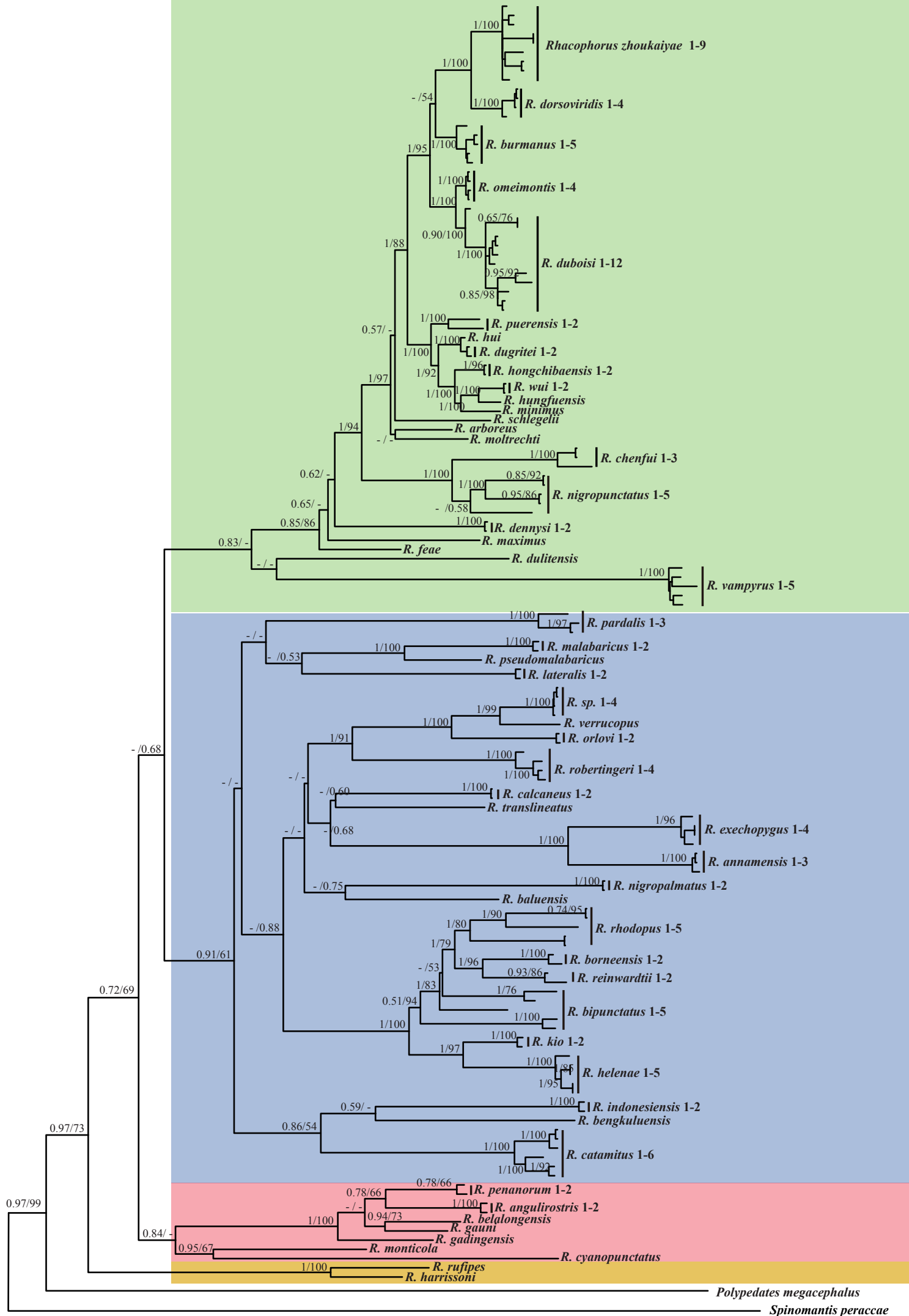

Supplement: Figure S1 — The nodal numbers are posterior probabilities ( >80% retained) and ML ( >50% retained). [file peerj-05-3995-s001.pdf]

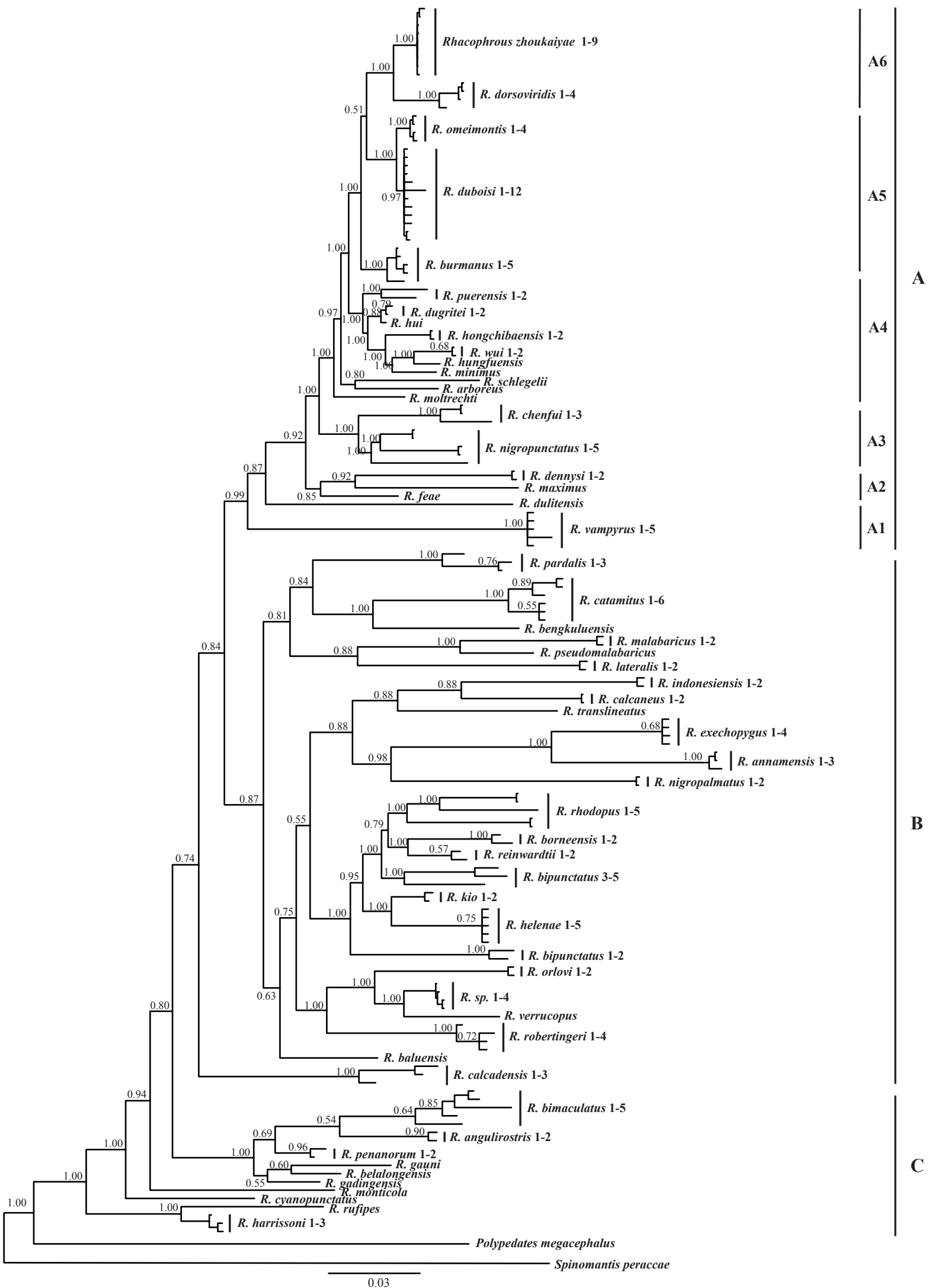

Supplement: Figure S2 — Group B and Group C were correspongding to the Clade B and Clade C in Fig. 2, respectively. [file peerj-05-3995-s002.pdf]
