# Supplementary material for: The reanalysis of biogeography of the Asian tree frog, Rhacophorus (Anura: Rhacophoridae): geographic shifts and climatic change influenced the dispersal process and diversification
Source: PeerJ. 2017 Nov 21;5:e3995. doi: 10.7717/peerj.3995 (PMC5701547; doi:10.7717/peerj.3995)
Supplement: Table S1 — “—” represents no molecular data. [file peerj-05-3995-s003.docx]

**Table S1. Samples, with sampling site, museum voucher nos., and GenBank accession nos. of corresponding sequences.** “---” represents no molecular data. The table was modified from Li et al. (2013).

| **Taxon** | **Locality** | **Voucher Number** |  |  |  | **Accession Number** |  |  |
| --- | --- | --- | --- | --- | --- | --- | --- | --- |
|  |  |  | **Partial 12S and 16S and complete t-RNA Val** | **BDNF** | **POMC** | **RAG-1** | **RHOD** | **TYR** |
| **Sub-order Anura** |  |  |  |  |  |  |  |  |
| **Family Mantellidae** |  |  |  |  |  |  |  |  |
| **I *Spinomantis*** |  |  |  |  |  |  |  |  |
| *S. peraccae*^1,2^ | Madagascar: Fianarantsoa, Ivohibe, Andringitra Volotsangana River | UMMZ 213278 | DQ283036 | --- | --- | --- | --- | --- |
| **Rhacophoridaae** |  |  |  |  |  |  |  |  |
| **II *Polypedates*** |  |  |  |  |  |  |  |  |
| *P. megacephalus* #^1,2^ | China | Genbank | AF458141 | --- | --- | --- | --- | --- |
| **III *Rhacophorus*** |  |  |  |  |  |  |  |  |
| *R. angulirostris*^1,2^ | Malaysia: Sarawak | ZRC 1.12076 | JN377348 | --- | --- | --- | --- | --- |
| *R. angulirostris*^1,2^ | Malaysia: Sabah, Bundu Tuhan | ZMH A13090 | JN377347 | --- | --- | --- | --- | --- |
| *R. annamensis*^1,2^ | Vietnam: Bu Gia Map National Park | KIZ 64 | JX219448 | --- | --- | --- | --- | --- |
| *R. annamensis*^1,2^ | Vietnam: Ta Kou Mountain Natural Reserve | KIZ 1196 | JX219446 | --- | --- | --- | --- | --- |
| *R. annamensis* #^1,2^ | Vietnam | AMNH A161414 | DQ283047 | --- | --- | --- | --- | --- |
| *R. arboreus* #^1,2^ | Japan | TTU-R-11748 | AF458142 | --- | --- | --- | --- | --- |
| *R. baluensis*^1,2^ | Malaysia: Sabah | FM235958 | KC961239 KC961089 | KC961093 | KC961153 | --- | --- | --- |
| *R. belalongensis*^1,2^ | Borneo: Brunei Darus-salam | ZMB70378 | JN705324 JN377352 | KC961101 | KC961144 | --- | --- | --- |
| *R. bengkuluensis*^1,2^ | Indonesia: Sumatra, Lampung | UTA A-62770 | KM212948 | --- | --- | --- | --- | --- |
| *R. bimaculatus* #^2^ | Philippines | RMB 10321 | KF933273 | --- | --- | --- | KF933204 | --- |
| *R. bimaculatus* #^2^ | Philippines | ACD:5395 | KF933272 | --- | --- | --- | KF933203 | --- |
| *R. bimaculatus* #^2^ | Philippines | ACD:4460 | KF933271 | --- | --- | --- | KF933202 | --- |
| *R. bimaculatus* #^2^ | Philippines | ACD:4350 | KF933270 | --- | --- | --- | --- | --- |
| *R. bimaculatus* #^2^ | Philippines | ACD:5798 | KF933269 | --- | --- | --- | --- | --- |
| *R. bipunctatus* #^1,2^ | China: Hainan | SN 030035 | EU215529 | --- | --- | --- | --- | --- |
| *R. bipunctatus* #^1,2^ | Vietnam | Genbank | AF458144 | --- | --- | --- | --- | --- |
| *R. bipunctatus*^1,2^ | Myanmar: Bee Hoe village, Chin State | CAS 235303 | JX219444 | --- | --- | --- | --- | --- |
| *R. bipunctatus*^1,2^ | Myanmar: Putao District, Kachin State | CAS 229913 | JX219445 | --- | --- | --- | --- | --- |
| *R. bipunctatus*^1,2^ | Vietnam: Ha Tinh, Huong Son District | AMNH-A 161418 | AY843750 | --- | --- | --- | --- | --- |
| *R. borneensis*^1,2^ | Malaysia:Sabah, Maliau Basin | BORN:22411 | AB781694 | --- | --- | --- | --- | --- |
| *R. borneensis*^1,2^ | Malaysia:Sabah, Maliau Basin | BORN:22410 | AB781693 | --- | --- | --- | --- | --- |
| *R. burmanus*^1,2^ | China: Motuo, Xizang | RAO 6239 | JX219422 | --- | --- | --- | --- | --- |
| *R. burmanus*^1,2^ | China: Mt. Gaoligong, Yunnan | SCUM 060614L | EU215537 | --- | --- | --- | --- | --- |
| *R. burmanus*^1,2^ | China: Gongshan, Yunnan | KIZ1049 | EF564497 EF564569 | --- | --- | --- | --- | --- |
| *R. burmanus*^1,2^ | China: Gongshan, Yunnan | KIZ1039 | EF564496 EF564568 | --- | --- | --- | --- | --- |
| *R. burmanus* #^1,2^ | China: Mt. Gaoligong, Yunnan | SCUM 060614L | EU215537 | --- | --- | --- | EU215567 | EU215597 |
| *R. calcadensis*^2^ | India: Kadalar, Idukki, Kerala | SDB.2011.291 | KC571276 | --- | --- | --- | --- | --- |
| *R. calcadensis*^2^ | India: Kalakkad Mundanthurai Tiger Reserve | SDBDU 2005 | KC571275 | --- | --- | --- | --- | --- |
| *R. calcadensis*^2^ | India: Kaikatti, Palaghat, Kerala | SDB.2010.257 | KC571274 | --- | --- | --- | --- | --- |
| *R. catamitus* #^1,2^ | Indonesia | UTA:A-60849 | JF748392 | --- | --- | --- | --- | --- |
| *R. catamitus* #^1,2^ | Indonesia | UTA:A-60848 | JF748391 | --- | --- | --- | --- | --- |
| *R. catamitus* #^1,2^ | Indonesia | UTA:A-60846 | JF748390 | --- | --- | --- | --- | --- |
| *R. catamitus* #^1,2^ | Indonesia | UTA:A-53998 | JF748389 | --- | --- | --- | --- | --- |
| *R. catamitus* #^1,2^ | Indonesia | UTA:A-59551 | JF748388 | --- | --- | --- | --- | --- |
| *R. catamitus* #^1,2^ | Indonesia | ENS 7657 | JF748387 | --- | --- | --- | --- | --- |
| *R. chenfui*^1,2^ | China: Zhaotong, Yunnan | RAO ZT 0806013 | JX219431 | --- | --- | --- | --- | --- |
| *R. chenfui*^1,2^ | China: Mt. Omei, Sichuan | Li05 | JX219432 | --- | --- | --- | --- | --- |
| *R. chenfui*^1,2^ | China: Mt. Omei, Sichuan | SCUM 060404L | EU215534 | --- | --- | --- | --- | --- |
| *R. calcaneus* ^1,2^ | Vietnam: Bi Doup National Park | KIZ 528 | JX219450 | --- | --- | --- | --- | --- |
| *R. calcaneus* ^1,2^ | Vietnam: Bi Doup National Park | KIZ 746 | JX219451 | --- | --- | --- | --- | --- |
| *R. cyanopunctatus*^1,2^ | Malaysia: Sarawak | NMBE 1056480 | KC961249 KC961084 | KC961098 | KC961152 | --- | --- | KC961230 |
| *R. dennysi*^1,2^ | China: Shaoguan, Guangdong | SCUM 060401L | EU215545 | --- | --- | --- | --- | --- |
| *R. dennysi*^1,2^ | China: Taoyuan, Hunan | Li06 | JX219433 | --- | --- | --- | --- | --- |
| *R. dorsoviridis*^1,2^ | China: Pingbian, Yunnan | RAO060821199 | JX219426 | --- | --- | --- | --- | --- |
| *R. dorsoviridis*^1,2^ | China: Jinping, Yunnan | RAO060821200 | JX219424 | --- | --- | --- | --- | --- |
| *R. dorsoviridis*^1,2^ | China: Pingbian, Yunnan | YN080446 | JX219425 | --- | --- | --- | --- | --- |
| *R. dorsoviridis*^1,2^ | Vietnam: Sa Pa, Lao Cai | ROM38015 | JX219423 | --- | --- | --- | --- | --- |
| *R. duboisi*^1,2^ | Vietnam: Sa Pa, Lao Cai | ROM 38758 | JX219414 | --- | --- | --- | --- | --- |
| *R. duboisi*^1,2^ | China:Lvchun County, Yunnan | LC0805088 | JX219417 | --- | --- | --- | --- | --- |
| *R. duboisi*^1,2^ | Vietnam: Sa Pa, Lao Cai | ROM 38771 | JX219413 | --- | --- | --- | --- | --- |
| *R. duboisi*^1,2^ | China: Jinping, Yunnan | RAOL060821289 | JX219415 | --- | --- | --- | --- | --- |
| *R. duboisi*^1,2^ | China: Lvchun County, Yunnan | LC0805089 | JX219416 | --- | --- | --- | --- | --- |
| *R. duboisi*^1,2^ | China: Jinxiu, Guangxi | RAOYN080492 | JX219412 | --- | --- | --- | --- | --- |
| *R. duboisi*^1,2^ | China: Mt. Dawei, Yunnan | YN080484 | JX219418 | --- | --- | --- | --- | --- |
| *R. duboisi*^1,2^ | China: Mt. Dawei, Yunnan | SCUM 061104L | EU215536 | --- | --- | --- | --- | --- |
| *R. duboisi*^1,2^ | Viet Nam:Lao Cai | VNMN:3217 | LC010603 | --- | --- | --- | --- | --- |
| *R. duboisi*^1,2^ | Viet Nam:Lao Cai | VNMN:4106 | LC010602 | --- | --- | --- | --- | --- |
| *R. duboisi*^1,2^ | Viet Nam:Lao Cai | VNMN:4105 | LC010601 | --- | --- | --- | --- | --- |
| *R. duboisi*^1,2^ | China: Jinping, Yunnan | KIZ060821289 | EF564495 EF564567 | --- | --- | EU924529 | EU924557 | EU924585 |
| *R. dugritei*^1,2^ | China: Hongya, Sichuan | SCUM 051017L | EU215540 | --- | --- | --- | --- | --- |
| *R. dugritei*^1,2^ | China: Baoxing, Sichuan | SCUM 051001L | EU215541 | --- | --- | --- | --- | --- |
| *R. dulitensis* #^1,2^ | Malaysia | RAO081201 | JX219434 | --- | --- | --- | --- | --- |
| *R. exechopygus*^1,2^ | Viet Nam:Gia Lai | VNMN:4108 | LC010586 | --- | --- | --- | --- | --- |
| *R. exechopygus*^1,2^ | Viet Nam:Gia Lai | VNMN:4107 | LC010585 | --- | --- | --- | --- | --- |
| *R. exechopygus* #^1,2^ | Viet Nam | ZFMK 86409 | GQ469980 | --- | --- | --- | --- | --- |
| *R. exechopygus* #^1,2^ | Viet Nam | ZFMK 86928 | GQ469979 | --- | --- | --- | --- | --- |
| *R. feae*^1,2^ | China: Hekou, Yunnnan | SCUM 050642WXJ | EU215544 | --- | --- | --- | --- | --- |
| *R. gadingensis*^1,2^ | Malaysia: Sarawak | NMBE 1057173 | KC961242 KC961087 | KC961102 | KC961145 | --- | --- | KC961223 |
| *R. gauni*^1,2^ | Malaysia: Sarawak, Bintulu Division | FMNH273928 | JX219456 | --- | --- | --- | --- | --- |
| *R. harrissoni*^2^ | Malaysia: Sarawak | NMBE 1056497 | JN705332 | KC961107 | KC961149 | --- | --- | KC961227 |
| *R. harrissoni*^1,2^ | Malaysia: Sarawak | NMBE 1057405 | JN705363 JN705330 | KC961105 | KC961147 | --- | --- | KC961225 |
| *R. harrissoni*^2^ | Malaysia: Sarawak | NMBE 1056492 | JN705331 | KC961104 | KC961148 | --- | --- | KC961226 |
| *R. helenae* #^1,2^ | Viet Nam | ZFMK:92544 | JQ288091 | --- | --- | --- | --- | --- |
| *R. helenae* #^1,2^ | Viet Nam | UNS:00451 | JQ288090 | --- | --- | --- | --- | --- |
| *R. helenae* #^1,2^ | Viet Nam | AMS:R 176399 | JQ288089 | --- | --- | --- | --- | --- |
| *R. helenae* #^1,2^ | Viet Nam | UNS:00450 | JQ288088 | --- | --- | --- | --- | --- |
| *R. helenae* #^1,2^ | Viet Nam | AMS:R 173230 | JQ288087 | --- | --- | --- | --- | --- |
| *R. hongchibaensis*^1,2^ | China: Wuxi, Chongqing | CIB 097687 | JN688883 | --- | --- | --- | JN688897 | JN688906 |
| *R. hongchibaensis*^1,2^ | China: Wuxi, Chongqing | CIB 097696 | JN688882 | --- | --- | --- | JN688898 | JN688907 |
| *R. hui*^1,2^ | China: Zhaojue, Sichuan, | Li01 | JN688878 | --- | --- | --- | --- | --- |
| *R. hungfuensis*^1,2^ | China: Wenchuan, Sichuan | SCUM 060425L | EU215538 | --- | --- | --- | --- | --- |
| *R. indonesiensis* #^1,2^ | Indonesia | MZB:Amp:23626 | AB983368 | --- | --- | --- | --- | --- |
| *R. indonesiensis* #^1,2^ | Indonesia | MZB:Amp:23619 | AB983367 | --- | --- | --- | --- | --- |
| *R. kio*^1,2^ | China: Xishuangbanna, Yunnan | SCUM 37941C | EU215532 | GQ285703 | GQ285734 | GQ285766 | EU215562 | EU215592 |
| *R. kio* #^1,2^ | Vietnam | Genbank | AF458147 | --- | --- | --- | --- | --- |
| *R. lateralis*^1,2^ | India: Bygoor, Karnataka | SDB.2010.330 | KC571277 | --- | --- | --- | --- | --- |
| *R. lateralis*^1,2^ | India:Mudigere | RBRL 050709-35, 36, 37 | AB530548 | --- | --- | --- | --- | --- |
| *R. malabaricus* #^1,2^ | India | Genbank | KR995135 | --- | --- | --- | --- | --- |
| *R. malabaricus*^1,2^ | India:Madikeri | Genbank | AB530549 | --- | --- | --- | --- | --- |
| *R. maximus*^1,2^ | China: Motuo, Xizang | RAO 6241 | JX219411 | --- | --- | --- | --- | --- |
| *R. minimus*^1,2^ | China: Mt. Dayao, Guangxi | KIZ 061214YP | EU215539 | --- | --- | --- | --- | --- |
| *R. moltrechti*^1,2^ | China: Lianhuachi, Taiwan | SCUM 061106L | EU215543 | --- | --- | --- | --- | --- |
| *R. monticola*^1,2^ | Indonesia: Sulawesi | RMB 1236 | AY326060 | --- | --- | --- | --- | --- |
| *R. nigropunctatus*^1,2^ | Vietnam: Sa Pa, Lao Cai | ROM 38011 | JX219427 | --- | --- | --- | --- | --- |
| *R. nigropunctatus*^1,2^ | China: Weining, Guizhou | GZ 070658 | JX219430 | --- | --- | --- | --- | --- |
| *R. nigropunctatus*^1,2^ | China: Longling, Yunnan | RAO 3496 | JX219428 | --- | --- | --- | --- | --- |
| *R. nigropalmatus* #^1,2^ | Malaysia | RAO081203 | JX219438 | --- | --- | --- | --- | --- |
| *R. nigropalmatus* #^1,2^ | Malaysia | RAO 081204 | JX219437 | --- | --- | --- | --- | --- |
| *R. nigropunctatus*^1,2^ | China: Longling, Yunnan | RAO 3494 | JX219429 | --- | --- | --- | --- | --- |
| *R. nigropunctatus*^1,2^ | China: Weining, Guizhou | SCUM 070657L | EU215533 | GQ285704 | GQ285735 | GQ285767 | EU215563 | EU215593 |
| *R. omeimontis*^1,2^ | China: Mt. Omei, Sichuan | SC 080505 | JX219421 | --- | --- | --- | --- | --- |
| *R. omeimontis*^1,2^ | China: Yaan, Sichuan | Li02 | JX219420 | --- | --- | --- | --- | --- |
| *R. omeimontis*^1,2^ | China: Pengxian, Sichuan | SCUM 0606137L | EU215535 | --- | --- | --- | --- | --- |
| *R. omeimontis*^1,2^ | China: Zhanotong, Yunnan | ZT 0806010 | JX219419 | --- | --- | --- | --- | --- |
| *R. orlovi*^1,2^ | China: Maguan, Yunan | RAO 03309 | JX219435 | --- | --- | --- | --- | --- |
| *R. orlovi*^1,2^ | Vietnam | AMNH A161405 | DQ283049 | --- | --- | --- | --- | --- |
| *R. pardalis*^1,2^ | Malaysia: Bintulu Division, Sarawak | FMNH 273243 | JX219454 | --- | --- | --- | --- | --- |
| *R. pardalis*^1,2^ | Malaysia: Bintulu Division, Sarawak | FMNH 273245 | JX219453 | --- | --- | --- | --- | --- |
| *R. pardalis*^1,2^ | Malaysia: Kota Marudu District, Sabah | FMNH 235741 | JX219452 | --- | --- | --- | --- | --- |
| *R. penanorum*^1,2^ | Malaysia: Sarawak | ZMH A10168 | JN377349 | KC961100 | KC961143 | --- | --- | KC961222 |
| *R. penanorum*^1,2^ | Malaysia: Sarawak | ZRC 1.12116 | JN377350 | --- | --- | --- | --- | --- |
| *R. pseudomalabaricus*^1,2^ | India: Kadalar, Kerala | SDB.2011.1010 | KC593855 | --- | --- | --- | --- | --- |
| *R. puerensis*^1,2^ | China: Puer, Yunnan | SCUM 060649L | EU215542 | --- | --- | --- | --- | --- |
| *R. puerensis*^1,2^ | Vietnam: Sa Pa, Lao Cai | ROM 37996 | JN688891 | --- | --- | --- | --- | --- |
| *R. reinwardtii*^1,2^ | Malaysia | RAO081205 | JX219443 | --- | --- | --- | --- | --- |
| *R. reinwardtii*^1,2^ | Malaysia:Johor, Endau Rompin | NNRn | AB728191 | AB728214 | AB728248 | AB728270 | AB728287 | AB728321 |
| *R. rhodopus*^1,2^ | China: Motuo, Xizang | RAO 06245 | JX219441 | --- | --- | --- | --- | --- |
| *R. rhodopus*^1,2^ | China: Lvchun, Yunnan | Lc0805109 | JX219440 | --- | --- | --- | --- | --- |
| *R. rhodopus*^1,2^ | China: Mengyang, Jinghong | SCUM 060692L | EU215531 | --- | --- | --- | --- | --- |
| *R. rhodopus*^1,2^ | China: Longchuan, Yunnan | Loc 08007018 | JX219439 | --- | --- | --- | --- | --- |
| *R. rhodopus*^1,2^ | China: Motuo, Xizang | RAO L062456 | JX219442 | --- | --- | --- | --- | --- |
| *R. robertingeri*^1,2^ | Viet Nam:Kon Tum | VNMN:3446 | LC010615 | --- | --- | --- | --- | --- |
| *R. robertingeri*^1,2^ | Viet Nam:Gig Lai | VNMN:4124 | LC010614 | --- | --- | --- | --- | --- |
| *R. robertingeri*^1,2^ | Viet Nam:Gig Lai | VNMN:4123 | LC010613 | --- | --- | --- | --- | --- |
| *R. robertingeri*#^1,2^ | Laos | FMNH 256465 | GQ204770 GQ204719 | --- | --- | GQ204600 | GQ204655 | --- |
| *R. rufipes*^1,2^ | Malaysia: Bintulu Division, Sarawak | FMNH 272858 | JX219455 | --- | --- | --- | --- | --- |
| *R. schlegelii*^1,2^ | Japan:Hiroshima | Genbank | AB202078 | --- | --- | --- | --- | --- |
| *R. sp.* ^1,2^ | China: Malipo, Wenshan, Yunnan | RAO 03308 | GQ285680 | --- | --- | --- | --- | --- |
| *R. sp.* ^1,2^ | China: Malipo, Wenshan, Yunnan | RAO 03324 | JX219408 | --- | --- | --- | --- | --- |
| *R. sp.* ^1,2^ | China: Malipo, Wenshan, Yunnan | RAO 03326 | JX219409 | --- | --- | --- | --- | --- |
| *R. sp.* ^1,2^ | China: Malipo, Wenshan, Yunnan | RAO 03321 | JX219410 | --- | --- | --- | --- | --- |
| *R. translineatus*^1,2^ | China: Motuo, Xizang | RAO 6237 | JX219449 | --- | --- | --- | --- | --- |
| *R. vampyrus* #^1,2^ | Viet Nam | AMS R 173132 | HQ656819 | --- | --- | --- | --- | --- |
| *R. vampyrus* #^1,2^ | Viet Nam | UNS 00104/AMS R 173129 | HQ656818 | --- | --- | --- | --- | --- |
| *R. vampyrus* #^1,2^ | Viet Nam | UNS 00103/AMS R 173128 | HQ656817 | --- | --- | --- | --- | --- |
| *R. vampyrus* #^1,2^ | Viet Nam | AMS R 173126 | HQ656816 | --- | --- | --- | --- | --- |
| *R. vampyrus* #^1,2^ | Viet Nam | NCSM77318 | HQ656815 | --- | --- | --- | --- | --- |
| *R. verrucopus*^1,2^ | China: Motuo, Xizang | 6254 RAO | JX219436 | --- | --- | --- | --- | --- |
| *R. wui*^1,2^ | China: Lichuan, Hubei | CIB 097685 | JN688881 | --- | --- | --- | JN688896 | JN688910 |
| *R. wui*^1,2^ | China: Lichuan, Hubei | CIB 097690 | JN688880 | --- | --- | --- | JN688900 | JN688909 |
| *R. zhoukaiyae* ^1,2^ | China: Jinzhai Country, Anhui | AHU-RhaDb-150418-01 | KU601494 | KU601449 | KU601458 | KU601467 | KU601476 | KU601485 |
| *R. zhoukaiyae* ^1,2^ | China: Jinzhai Country, Anhui | AHU-RhaDb-150418-02 | KU601495 | KU601450 | KU601459 | KU601468 | KU601477 | KU601486 |
| *R. zhoukaiyae* ^1,2^ | China: Jinzhai Country, Anhui | AHU-RhaDb-150418-03 | KU601496 | KU601451 | KU601460 | KU601469 | KU601478 | KU601487 |
| *R. zhoukaiyae* ^1,2^ | China: Jinzhai Country, Anhui | AHU-RhaDb-150418-04 | KU601497 | KU601452 | KU601461 | KU601470 | KU601479 | KU601488 |
| *R. zhoukaiyae* ^1,2^ | China: Jinzhai Country, Anhui | AHU-RhaDb-150419 | KU601498 | KU601453 | KU601462 | KU601471 | KU601480 | KU601489 |
| *R. zhoukaiyae* ^1,2^ | China: Jinzhai Country, Anhui | AHU-RhaDb-150420-03 | KU601499 | KU601454 | KU601463 | KU601472 | KU601481 | KU601490 |
| *R. zhoukaiyae* ^1,2^ | China: Jinzhai Country, Anhui | AHU-RhaDb-150420-01 | KU601500 | KU601455 | KU601464 | KU601473 | KU601482 | KU601491 |
| *R.* *zhoukaiyae* ^1,2^ | China: Jinzhai Country, Anhui | AHU-RhaDb-150420-02 | KU601501 | KU601456 | KU601465 | KU601474 | KU601483 | KU601492 |
| *R. zhoukaiyae* ^1,2^ | China: Jinzhai Country, Anhui | AHU-RhaDb-120428 | KU601502 | KU601457 | KU601466 | KU601475 | KU601484 | KU601493 |

# Speciman with no detail sampled sites; 1 Dataset 1; 2 Dataset 2.
