# Supplementary material for: The reanalysis of biogeography of the Asian tree frog, Rhacophorus (Anura: Rhacophoridae): geographic shifts and climatic change influenced the dispersal process and diversification
Source: PeerJ. 2017 Nov 21;5:e3995. doi: 10.7717/peerj.3995 (PMC5701547; doi:10.7717/peerj.3995)
Supplement: Table S2 [file peerj-05-3995-s004.docx]

**Table S2. Primers used in PCR and sequencing.**

| Locus | Primer | Primer sequence | Size (bp) | Cited source |
| --- | --- | --- | --- | --- |
| Recombination activating gene 1 | L6300 | 5’-CTG GTC GTC AGA TCT TTC AGC-3’ | 1164 | Li et al. (2009) |
|  | H6301 | 5’-GCA AAA CGT TGA GAG TGA TAA C-3’ |  | Li et al. (2009) |
| Proopiomelanocortin | R7120 | 5’-TAY TGR CCC TTY TTG TGG GCR TT-3’ | 601 | Wiens et al. (2005) |
|  | L7121 | 5’-GGA RCA CTT YCG ATG GGG YAA ACC-3’ |  | Wiens et al. (2005) |
| Brain-derived neurotrophic factor | R7151 | 5’-CTA TCT TCC CCT TTT AAT GGT C-3 | 614 | Vieites et al. (2007) |
|  | L7152 | 5’-ACC ATC CTT TTC CTT ACT ATG G-3’ |  | Van der Meijden et al. (2007) |
| Exon 1 of rhodopsin | L2903 | 5’-ACC ATG AAC GGA ACA GAA GGY CC-3’ | 315 | Bossuyt and Milinkovitch (2000) |
|  | H2904 | 5’-GTA GCG AAG AAR CCT TCA AMG TA-3’ |  | Bossuyt and Milinkovitch (2000) |
| Exon 1 of tyrosinase | L2976 | 5’-TGC TGG GCR TCT CTC CAR TCC CA-3’ | 531 | Bossuyt and Milinkovitch (2000) |
|  | H2977 | 5’-AGG TCC TCY TRA GGA AGG AAT G-3’ |  | Bossuyt and Milinkovitch (2000) |
